# Striving to Die: Medical, Legal, and Ethical Dilemmas Behind Factitious Disorder

**DOI:** 10.7759/cureus.13243

**Published:** 2021-02-09

**Authors:** Akriti Sinha, Trenton Smolik

**Affiliations:** 1 Psychiatry, University of Missouri Health Care, Columbia, USA; 2 Internal Medicine, CentraCare Hospital, St. Cloud, USA

**Keywords:** factitious disorder, munchausen's disorder, medically unexplained physical symptoms

## Abstract

Factitious disorder (FD) imposed on self is one of the most challenging and controversial problems in medicine. It is characterized by falsified medical or psychiatric symptoms where patients misrepresent, simulate, or cause symptoms of an illness in the absence of obvious tangible gains. Munchausen syndrome accounts for approximately 10% of all factitious illnesses and represents its most malignant form. An unknown number of deaths have likely occurred when considering that most cases go unrecognized and unreported. Here we describe a case in which the patient’s condition remained unrecognized, only being diagnosed months before her death from complications of FD.

Psychiatry was consulted to see a 49-year-old Caucasian female regarding depression, poor oral intake, and her insistence on the placement of a feeding tube. The initial evaluation was negative for findings consistent with psychiatric illness. A review of records in our hospital was significant for one previous psychiatric inpatient stay eight months prior during which a diagnosis of FD imposed on self was made. Collateral information suggested a cycle of deception and simulation of illnesses with the patient’s daughter labeling her actions as “doctor shopping.” At our facility alone, she had accrued roughly 40 inpatient medical admissions and 70 ED visits in four years though only two encounters involving Psychiatry. A detailed chronological analysis of her records showed the only documented concern of deception to be that of an Internal Medicine resident two years prior. Psychiatry was not consulted despite this concern. During the present encounter, psychiatry recommended ethics consult, outpatient psychotherapy, and frequent follow-ups with primary care. A formal ethics consult was not completed before discharge. Within two months, the patient died at another facility.

FD can lead to diagnostic and therapeutic procedures that result in irreversible morbidity and iatrogenic harm. Physicians in other medical specialties often suspect a patient of consciously deceiving others, though fail to assign psychiatric nomenclature due to lack of familiarity or comfort in making the diagnosis. This further substantiates the role of a multidisciplinary collaboration between medical, surgical, and psychiatry teams. Heightened awareness of, and suspicion for, Munchausen syndrome may improve rates of diagnosis and prognosis of these patients.

## Introduction

Factitious disorder (FD) involves falsification of psychological or physical signs or symptoms; induction of injury or disease associated with identified deception; and presentations of oneself as ill, injured, or impaired in the absence of obvious external rewards [1].

Despite recent attempts at improved classification, FD continues to be one of the most controversial and challenging problems in medicine [2]. Most healthcare providers will encounter at least one case throughout their practice [3]. The prevalence of FD imposed on self is difficult to estimate because of the evasive nature of the disorder. If counseled, patients are prone to deny the illness and seek care elsewhere further complicating their treatment. Multiple reviews estimate the incidence of FD at 1% though, in specific healthcare settings, it can be higher [4]. A systematic review by Yates et al. showed that 66.2% of patients with FD were female, the mean age at presentation was 34.2, and over half of patients had a job in healthcare [5]. Munchausen syndrome accounts for approximately 10% of all factitious illnesses and represents the most malignant form [6]. Numerous deaths from this condition have been reported while more go unrecognized and unreported [7,8]. Annual healthcare costs in individual cases of FD have been up to $1,000,000 [9]. Here we describe a case in which the patient remained undiagnosed for years, only being formally recognized months before her death from complications of FD.

## Case presentation

Our psychiatry team was consulted on a 49-year-old Caucasian female for concerns of possible depression and medication non-compliance. She had been admitted to the hospital with chief complaints of dizziness, malaise, decreased appetite, and inability to care for herself at home. Concern for intentional refusal of food intake had been raised, and the patient was insistent upon feeding tube placement. Intact colostomy, central IV port, and urostomy were present at admission. The workup for her above issues was unremarkable and the patient had been educated extensively on nutrition.

During psychiatric consultation, the patient discussed an inability to keep food down since a revision colostomy procedure two months ago, resulting in the loss of 70 pounds. She felt neglected and victimized by doctors, fixating on their reluctance to address her pain or insert a feeding tube. Inconsistencies in her medical history were attributed by the patient to poor memory. Health issues were reported to have begun after a rectal prolapse 10 years prior. For the past four years, she had been on disability along with Medicare A and B plans. She had recently separated from her boyfriend. Her eldest daughter, also her durable power of attorney, was professed to just be after money despite the patient’s self-professed limited finances. She denied having social relationships outside of her children. Psychiatric history was denied. Examination revealed an ill-appearing female with a colostomy and a urostomy bag. Her abdomen exhibited multiple scars best characterized as a “grid-iron abdomen”. Due to a request from the patient to cease talking, the interview was ended prematurely. 

A thorough evaluation of her medical record in our hospital identified extensive surgical history including thyroidectomy, appendectomy, hysterectomy, oophorectomy, cholecystectomy, colostomy formation, as well as multiple exploratory laparotomies with adhesiolysis in the past decade in unknown facilities. Medically complex, she also was denoted to have chronic abdominal pain, Hashimoto thyroiditis, recurrent urinary tract infections, and insulin-dependent diabetes mellitus. Despite her claim of no psychiatric history, the patient was being prescribed clonazepam, duloxetine, escitalopram, and Ambien from her primary care provider. Due to the extensive utilization of multiple healthcare facilities, a comprehensive chart review of external records was unable to be performed.

Totaling nearly 40 inpatient admissions and 70 ED visits, she had spent almost 100 days as an inpatient between 2014 and 2018 in our hospital alone (Table 1). Known to providers in multiple clinics; Vascular Surgery, Urology, Uro-Gynecology, Surgical-Oncology, Plastic Surgery, PM&R, Otolaryngology, Orthopedics, Ophthalmology, Neurology, Nephrology, Internal Medicine, Infectious Disease, General Surgery, Gastroenterology, Endocrinology, Dermatology, and Cardiology, she had been extensively evaluated for a variety of issues. Of all these providers, only a single medical resident had expressed concern about potential fabrication of illness two years prior though they neglected to consult the psychiatry team. 

**Table 1:** Chronology of admissions of the patient in our hospital SNF: Skilled Nursing Facility, UTI: Urinary Tract Infection, ENT: Ear Nose Throat, TPN: Total Parenteral Nutrition, IVF: Intravenous Fluids, PT: Physical Therapy, OT: Occupational Therapy, TIA: Transient Ischemic Attack

Duration of Stay, Primary Team	Symptoms/Chief Complaint	Tests	Outcome, Plan	Consults	Comments
6/2014 (1 day) Internal Medicine	Dysuria, Dehydration Recurrent UTI's in that past with multi-resistant bacteria, increased ostomy output, rectal prolapse	Concerns of Cystitis on Urinalysis	Discharge on Levofloxacin Follow Up with Surgery, Primary Care	ENT for Otitis Externa	Recently moved here from outside state and is in the process to establish care
9/2014 (1 day) Surgical-Oncology	Rectal Pain Concern for Abscess	Admitted for pain control, IV fluids and antibiotics. No evidence of abscess or erythema	Discharged on Oxycodone, Ciprofloxacin Follow up with Surgery	None	
12/2014 Emergency Department	Anaphylactic reaction after receiving a cervical epidural injection chronic radiculopathy		Discharge on Benadryl, EpiPen, Famotidine, Oxycotin, Predinsone	None	
5/2015 (3 days) Surgical- Oncology	Leakage at colostomy Sites/Dysfunctional Ileostomy	1. Exploratory laparotomy with resection of old colorectal anastomosis. 2. Creation of end colostomy. 3. Closure of ileostomy.	Home with Assistance and Home Health	None	
5/2015 (1 day) Surgical- Oncology	1. Colostomy Obstruction 2. Wound Infection	Conservative Management			
6/2015 (2 days) Surgical-Oncology	Bilious vomiting and low ostomy output.	Aggressive bowel regimen.	Follow up with Surgery	None	Noncompliant with regimen in the past.
12/2015 (1 day)	Scheduled subcutaneous port removal				
12/2015 (1 day) Gastroenterology	Heart burn and pain radiating to the right side	Esophagogastroduodenoscopy (EGD)			
7/2016 (2 Days) Cardiology	Chest Pain, Diarrhea States history of abnormal EKGs which were suggestive of previous Myocardial Infarction	Given aspirin 324mg and nitro sublingual x 1 EKG showed sinus rhythm with Troponins were negative. Negative exercise stress test. No coronary angiography was performed	She was discharged home with cardiology follow-up.	Observation by treating resident: Patient has received antibiotics in the past few weeks including Bactrim, vancomycin, ciprofloxacin and metronidazole. It implausible for her to have cystitis in light of the vast amount of antibiotics she has been given. She stated that she did not actually take all of these antibiotics but she did have them filled. She made mention of several recent doctor and ED visits both within and outside of this health system. In her history, she has had multiple different surgeries and has a long list of medical diagnoses. While I believe that she has multiple chronic conditions, I am concerned that these are exacerbated by underlying psychiatric disease and should be evaluated as an outpatient by a psychiatrist.	
8/2016 Cardiology	Patient presents for: Elective Cardiac Cath, Coronary Angiography and possible Intervention	No procedure needed			
1/2017 (2 days) Internal Medicine	Recurrent Clostridium difficile diarrhea	Was given oral Vancomycin Tapering regimen for 6 weeks	Surgery consulted; No acute surgical intervention and outpatient follow up.	Follow up with Urology, Primary care	None
3/2017 (2 days) Internal Medicine	Abdominal Pain with increase in colostomy output and subjective fever for the past 3 days	Clostridium difficile positive. Port placement	Discharge on fidaxomicin General surgery and Infectious Disease Follow Up	Infectious Disease Interventional Radiology General surgery	Pt requested neurology follow up for bell’s palsy and endocrine follow up 2 falls in recent months. Rrecommended wheeled walker but she is declining as she states her home is too small for a walker
6/2017 Emergency Department	Abdominal Pain	No indication for Surgery Pain control with oxycodone		Surgery-Oncology	
7/2017 (6 Days) Internal Medicine	Abdominal Pain Code Stroke	Acute onset of Left facial weakness and Left lower leg weakness that resolved within 24 hours, she had TPA administered.	Noted to have no residual infarcts or hemorrhages on MRI. Discharge to facility	Neurology	
9/2017 (6 Days) Surgery-Oncology	Colonic inertia, Anal stricture, Parastomal hernia, Ventral hernia	Partial colectomy, colostomy takedown, creation new ostomy, anal dilation and sphincterotomy	Discharge to Facility		
11/2017 (2 Days) Internal Medicine	Dehydration Orthostatic Hypotension	IVF, patient remained hemodynamically stable and electrolytes normal	Patient was discharged in stable condition to follow up with Primary	Pt initially requested TPN but was educated about the needs for TPN and the associated risks. She requested IV fluids at home due to concerns about becoming dehydrated although she has been tolerating PO since admission. She informed the treatment team that her home situation is complicated by several things. She says her daughter is mentally incapacitated and the daughter's father is a predator.	
1/2018 Emergency Department	Dysuria	No signs of infection identified. Foley catheter was placed for retention	Follow Up with urology service for further management		
5/2018 (2 Days) Internal Medicine	Dysuria		Explained that oxycodone is not good for urinary retention	Patient non-compliant to antibiotic	
6/2018 (4 Days) Internal Medicine	Persistent Dysuria	Started on Ceftriaxone for suspected UTI. Urology found no cause for urgent urological intervention		Urology	
6/2018 (8 Days) Urology	Recurrent UTI and Urinary Retention	Ileal Conduit Postoperative course was notable for Hypotension, Hypoxemia, Anemia, Leukocytosis, Thrombocytopenia, Hypocalcemia and Hypomagnesemia.	Discharge to SNF		
6/2018 916 Days) Internal Medicine	Fever, Chills Increased liquid output in stool consistency in ileostomy which has occasionally been 'black'	Urology reports no intervention at this time; doubt post-operative infection,	Reports she felt "chills". Iinstructions to follow-up for a CT Discharge to SNF	Urology Interventional Radiology	Refusal to take potassium supplementation due to concerns of dysphagia with tablets. Also has been refusing insulin and accuchecks. Interested in switching her tramadol to oxycodin
7/2018 (7 Days) Internal Medicine	Abdominal Pain	CT abdomen concerning for signs of peritonitis, possible new abscess formations, and hydronephrosis. Cr elevated Started on vancomycin and ertapenem+ Micafungin	Discharge to Facility	Infectious Disease Gastroenterology Interventional Radiology	
7/2018 (4 Days) Gastroenterology	Chronic Anemia For EGD	ENDOSCOPIC DIAGNOSIS: Gastropathy. Duodenal erosion.			
8/2018 (1 Day) Internal Medicine	“Unable to manage care"	PT recommendations prior to that discharge patient to go to SNF for rehab and assistance with care. However, patient refused SNF and was discharged home on IV daptomycin and ertapenem. Patient states she was set up with home health care and home health nurse. However, soon after the nurse left the patient needed assistance and her daughter was unable to help. She was too weak to rise from toilet and had to call home health nurse to come back for assistance. She realized she needs more help than she is able to get at home and returned to the ED for help with placement to SNF.			
8/2018 (10 Days) Internal Medicine	Dizziness, malaise, Decreased appetite	TSH 24.6, T4 0.62. Urinalysis is concerning for UTI and reflex urine culture shows gram negative rods likely from gut flora due to urostomy ileo conduit. -extensive nutrition counseling -PT consult -Consider hospice -Psychiatry consult on depression re-evaluation and med recs	Patient was educated extensively on nutrition but requested G tube	Discharge to home	Pt was diagnosed with Factitious Disorder by Psychiatry and ethics was recommended to be consulted; not performed by primary.
9/2018 (8 Days) Internal Medicine	Weight loss Recurring bacterial infection Decreased vision, "left optic nerve affected due to malabsorption." "Polyps in stomach" found by endoscope 2 months ago. Has "TIAs" frequently	Per nursing, wants feeding tube.	PT worked with patient, who walked 5 feet and could not go any further due to numbness of thighs and feet; patient requested cardiology consult as she thinks these symptoms are caused by her heart.	Per nursing, legs were numb overnight, but patient flinched when nursing touched her legs. Per patient, recently kicked out of house by daughter. Patient refused PT/OT, who recommend discharge home	

Her only prior psychiatric contact in our hospital was eight months prior when she had reported suicidal ideation after the Emergency Department (ED) refused to administer further pain meds. This led to her admission at our psychiatric inpatient unit on a voluntary status during which she demanded to be put on total parenteral nutrition and intravenous fluids despite a lack of indication. During this stay, every five-minute blood sugar checks had been requested and she sought a very tailored insulin regimen. Appearing to deliberately struggle during cognitive testing, she seemed disappointed to have not been diagnosed with dementia. Social history obtained was significant for the patient living with her daughter who was also her durable power of attorney (DPOA). The patient had graduated from high school and had a non-medical associate degree. She had been married and divorced three times. She had experienced physical and verbal abuse in childhood and sexual abuse as an adult. She denied substance abuse. Her family history was significant for schizophrenia in the father and alcohol use in the brother.

Collateral from her daughter during inpatient stay revealed history consistent with FD ongoing for several years. A prior boyfriend, who was an emergency medical technician, had departed due to frustrations about her tendency to do the opposite when given medical advice. It was also learned that the patient would present to the ED up to three times weekly simply to get "checked out." Unprompted, her daughter identified that she “doctor shopped” for diagnoses and treatment to her liking, such as undergoing an elective colectomy for abdominal pain. During this first contact with our psychiatry team, she was diagnosed with severe FD imposed on self and was encouraged to start seeing Psychiatry and her Primary Care more frequently. None of the recommendations were followed by the patient.

Now with this second contact, collateral was again obtained from the daughter revealing further induction and fabrication of illness. Among her actions were drinking essential oils, self-induced dehydration, purposeful contamination of her stoma with bacteria, as well as non-compliance to antibiotics. Seeing providers spanning multiple states, she had accumulated several needless elective procedures and would swiftly switch doctors should they refuse her. Not satisfied with the care in Missouri, she tentatively planned to seek care in Texas. Past providers had suggested feigning symptoms, but she was never diagnosed nor treated for FD. Her daughter summarized that she had never tried to get better.

In the light of falsification of physical signs or symptoms, induction of injury or disease, with identified deception evident in the absence of obvious external rewards and the absence of psychosis or delusions, we diagnosed FD imposed on self with recurrent episodes consistent with the Diagnostic and Statistical Manual of Mental Disorders, fifth edition (DSM‐V) diagnostic criteria. Given the severity of her medical illness, multiple recent admissions, and high rates of healthcare utilization, we emphasized routine follow-ups with Primary Care, initiation of Psychotherapy, and consultation with the Ethics team. Unfortunately, the patient was discharged the next day without a formal ethics consultation or confrontation by the providers.

Directly opposing her mother, the patient's daughter insisted she follows up with a psychiatrist but was refused. While her daughter attempted to gain guardianship, the patient passed two months later at an unaffiliated hospital after a protracted hospitalization with multiorgan failure and septic shock.

## Discussion

Medical implications 

In 1951, the term “Munchausen syndrome” first appeared in a Lancet article by Asher comparing the discussed patients to the famous braggart Baron von Munchausen in that, both of their stories were dramatic and untruthful [10]. Famed psychiatrist Theodore Nadelson felt it nigh inevitable that patients with Munchausen syndrome would eventually cross the line into malingering, recognizing there can be tangible rewards attained when deceiving a physician. Barker, a British psychiatrist, suggested reclassification of Munchausen disorder as hospital addiction [11]. 

Often inappropriately discussed as having “thick chart syndrome” or termed “black hole patients” the prevalence of those with FD goes under-reported as they remain undiagnosed even when highly suspected. FD imposed on self is commonly associated with psychosocial factors, including early losses via death or abandonment; disrupted attachments to caregivers due to abuse or institutionalization, gratifying experiences related to the sick role, and a desire for attention. It is hypothesized that the sick role behavior associated with FD is a means of establishing one's identity, maintaining relationships with others, and addressing emotional dysregulation and unmet needs [4].

Commonly psychiatric comorbidities confound diagnosis, with a retrospective study of FD patients showing other psychiatric comorbidities to be present in more than 30%, with a history of physical or sexual abuse reported by more than 20%. Increased rates of depression (41%), anxiety (14%), and personality disorders (16.5%), particularly borderline personality disorders, substance abuse (15.3%), functional neurological symptoms (5.3%), and eating disorders (4.1%) are diagnosed in FD patients. A total of 14.1% of patients reported current suicidal ideation or a history of suicide attempts [5].

Not infrequently, the patient leaves against medical advice or is discharged before psychiatric or ethical consultation is obtained, leading to an absence of longitudinal follow-up, as seen in our case [8,12]. Though not infrequently suspected by physicians, psychiatric nomenclature is seldom assigned due to a lack of familiarity or comfort in making the diagnosis. Most physicians recognize when they are being deceived, though may not know the formal difference between malingering, FD, and somatoform disorders [8]. Demands and aggravation by a patient can cause even a seasoned practitioner to dispense addictive medications or perform procedures ranging from unnecessary to dangerous. Frustrations with these patients can yield bitter arguments, punitive confrontations, and missed diagnoses of true medical illnesses [13]. Since these patients often seek care at multiple hospitals, there is often missing information that prevents accurate recognition leading to inappropriate procedures, as seen in our case.

Effectively any illness can be simulated or induced though the most commonly falsified symptoms and diseases include abdominal pain, arthralgia, chest pain, coagulopathy, diarrhea, hematuria, hypercortisolism, hyperthyroidism, hypoglycemia, infections, seizures, and non-healing skin wounds. Professionals working in Endocrinology, Cardiology, and Dermatology are the most susceptible to encounters with these patients. Patients with FD often incline towards “fast-track” admissions, such as due to stroke and retrosternal chest pain (Table 1) [5].

Ethical implications

Feeling deceived evokes strong feelings of countertransference amongst healthcare workers. Even if willing to access help, a factitious patient may have a difficult time finding a therapist. The lack of awareness surrounding FD lends to an environment in which few therapists can fully respond to the unique emotional needs of both these patients and the providers who encounter them [14]. Discussion of the diagnosis with the patient is appropriately termed confrontation and various techniques are described ranging from the hard-hitting approach, to the gentle, persuasive confrontation, down to the subtle approach [15]. Though most FD patients deny inducing their illness when confronted, this reality check approach is needed as the patient may have very poor insight into the severity of their condition. Our patient was never directly confronted by her primary team or by Psychiatry and continued to fabricate illness allowing her prognosis to worsen. 

A textbook case of how FD can lead to morbidity and iatrogenic harm, our patient underwent unnecessary diagnostic and irreversible therapeutic procedures for years before establishing care in our hospital. Particularly among patients identified late in their course, recovery is infrequent. A case series of 20 patients found that factitious behavior directly led to the deaths of 20 percent. One patient with FD admitted to having undergone 42 surgical procedures over the course of 850 admissions at 650 different hospitals [16]. Undiagnosed FD allows a futile cycle of care to persist, to the detriment of the patient and the healthcare system.

Principles of management include clear communication and collaboration amongst all professionals involved in the patient’s care, and early involvement of Psychiatry and Ethics teams. Unfortunately, these efforts are often confounded by a patient’s tendency to seek care at multiple unaffiliated hospitals. 

The patient discussed herein never received an ethics consult, likely due to her late diagnosis, poor prognosis, and the opinion that such a consult might not be considered necessary in the late stage as they may appear beyond the window for psychotherapy. Doctors need to be taught conceptual, developmental, and management frameworks to understand and treat these patients. Firm boundaries are needed to reduce distress in both staff and the patient. To a fault, medical education leaves providers ill-prepared for these occasions in which confrontation is not only appropriate but encouraged [17]. 

Legal implications

As was in our case, noncompliance with treatment is a major feature of this illness. A review of 13 observational studies (total n = 284 patients) found that more than 60% either refused or did not follow up with psychiatric treatment [18]. Patients commonly threaten lawsuits, become disruptive, leave against medical advice, or simply seek care elsewhere. Clinicians may become defendants in litigation initiated by family members of patients who die as a result of FD [14]. A particularly egregious case described a young woman who after feigning cancer later sued 35 physicians collectively for $14 million [19]. 

Beyond these already convoluted ethical implications, providers are led to the risky medicolegal territory when treating these patients. Negotiating the complex framework of protections like the Health Insurance Portability And Accountability Act (HIPAA) further encumbers the care of FD patients. Although justifiable to prevent iatrogenic injuries, obtaining external records without consent is contentious. Legal advice should be sought before acting on such requests due to the inherent complexity. Arguments in favor of a national registry of factitious patients to help monitor and curb their excessive use of healthcare have been raised, but this idea remains controversial. Detractors raise concerns that every person on such a list would receive substandard care, or no care at all in the event of a genuine medical problem. Moreover, there is the likelihood that everyone on such a registry would be promptly and permanently denied health insurance [14]. Covert video surveillance (CVS) and room searches are commonly considered invasions of privacy and are not universally implemented. 

In most states, involuntary commitment is limited to patients with imminent suicidal risk, of overt danger to others, or lacking the capacity to care for their basic needs. FD does not fulfill these requirements. Few cases in which a patient has been committed solely based on FD, Munchausen syndrome, and/or malingering exist in the United States. Oregon has a unique alternative to commitment, using a medical conservatorship under which a guardian is appointed to make all medical decisions on behalf of the patient [20].

## Conclusions

This case illustrates how patients with lethal FD or Munchausen syndrome have a protracted course, complicated by repeated hospitalizations, ultimately leading to their premature deaths. As was true of our patient, few are referred to psychiatric services and even fewer accept care. Clinicians should be particularly vigilant for FD in patients who are female, in early adult life, or have worked in healthcare. Although patients with FD may appear in any specialist setting, Endocrinology, Cardiology, and Dermatology services should be particularly mindful. Our case report further substantiates the role of an early multidisciplinary collaboration between Medical, Surgical, Ethics, and Psychiatry teams to improve rates of diagnosis and treatment. Psychiatric comorbidities should be identified and treated in a timely fashion. The differential diagnosis for FD is broad and includes not only organic disorders but also somatic symptom disorder, malingering, conversion disorder, and borderline personality disorder. Clear guidelines on the management of these patients need to be set to protect both patients and providers in light of the ethical and legal considerations. States must consider existing laws in the context of these patients and provide legal capacity to use involuntary commitment and appointment of medical conservatorship when needed. Beyond the personal obligations of physicians, an additional impetus is on both the educational and regulatory bodies of medicine to help equip providers to care for these uniquely challenging patients. 
